# Supplementary material for: Chemogenetic attenuation of cortical seizures in nonhuman primates
Source: Nat Commun. 2023 Feb 28;14:971. doi: 10.1038/s41467-023-36642-6 (PMC9975184; doi:10.1038/s41467-023-36642-6)
Supplement: Supplementary file 1 — Supplementary Information [file 41467_2023_36642_MOESM1_ESM.pdf]

Supplementary Information

Supplementary Table 1. Summary of treatment conditions and clonic seizure magnitude

Subject	Session#	Bicuculline (μg)	Vehicle	interval-1 (min)	1st DCZ (mg/kg)	interval-2 (min)	2nd DCZ (mg/kg)	Normalized clonic seizure
Monkey #1	1	8	-	-	0.1	-	-	-1
	2	16	✓	36	0.1	18	0.1	-0.45
Monkey #2	1*	0.4	-	-	0.1	30	0.1	-
	2 (contra)	0.4	-	-	0.1	29	0.1	-
	3	4	✓	15	0.1	30	0.1	-0.74
	4	1	✓	15	0.1	30	0.1	-1
	5	1.4	✓	15	0.1	30	0.1	-1
	6 (contra)	4	✓	15	0.1	30	0.1	-

Check marks indicate the session in which the subject received vehicle injection. Interval-1 indicates the time difference between bicuculline and the 1st DCZ injection, and interval-2 indicates the time difference between the 1st and 2nd DCZ injections. *In this session, the video data was accidentally lost and was excluded from the analysis of clonic seizures. Note that although the dose of bicuculline differed from session to session, there was no significant effects of bicuculline dosage on chemogenetic action in either cortical seizure (linear regression analysis, $F_{(1,4)} = 3.06$, $p = 0.16$) or clonic seizures ($F_{(1,3)} = 5.64$ $p = 0.098$).

Supplementary Table 2. Summary of focal-to-bilateral seizure sessions in monkey #2

Subject	Session#	Bicuculline (μg)	Waveforms before 1st DCZ (–5~0 min)		1st DCZ (mg/kg)	Waveforms after 1st DCZ (5~10 min)	
			Ipsilateral	Contralateral		Ipsilateral	Contralateral
Monkey #2	1	0.4	MSWC, SE	MSWC	0.1	S	S
	3	4	MSWC, rSE	MSWC, rSE	0.1	MSWC, SE	MSWC
	4	1	MSWC, rSE	MSWC, rSE	0.1	S	S
	5	1.4	MSWC, rSE	MSWC, SE	0.1	SWC	S

S, Spikes; *SWC*, Spike-wave complex; *MSWC*, Multi spike-wave complex; *SE*, Status epilepticus; *rSE*, Refractory SE; Severity of seizures is given in the order S < SWC < MSWC < SE < rSE. Examples of waveforms are shown in Fig. 2a and Supplementary Fig. 8c-e.

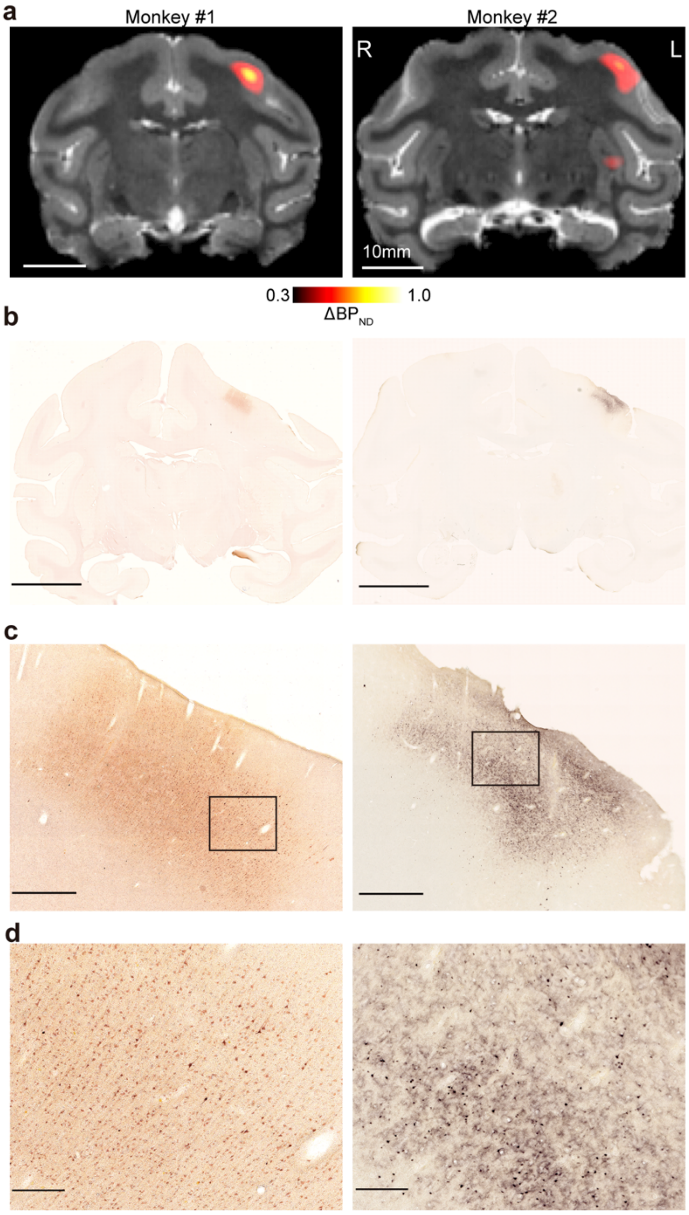

Supplementary Figure 1. Comparison of DREADD expression between monkeys. **a**. Coronal PET images from monkeys #1 (left) and #2 (right) in which [^{11}C]DCZ is co-registered with an MR image showing *in vivo* hM4Di expression in MI. **b-d**. Anti-GFP immunohistochemical staining for monkeys #1 (left) and #2 (right). Left and right black boxes in **c** are respectively enlarged in **d**. Images were digitally enhanced by redefining the tone curves linearly. Scale bars. **a** and **b,** 10 mm; **c**, 1 mm; **d**, 250 µm. The results shown are representative for the two subjects.

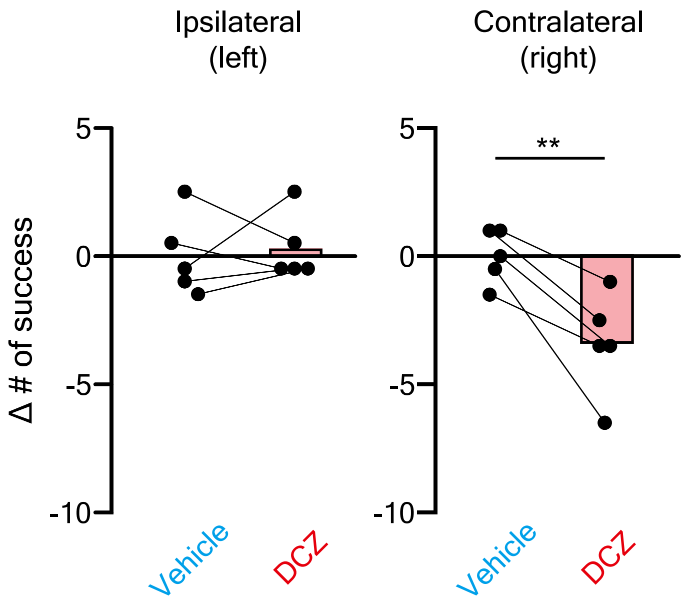

Supplementary Figure 2. Effect of hM4Di activation on precision grip capability. Treatment-induced change in the number of successful food-pellet retrievals by monkey #2 in the modified Brinkman-board task using hands ipsilateral (left) and contralateral (right) to the hM4Di-expressing hemisphere. DCZ application significantly affected the right hand (p = 0.0097, two-tailed paired t test, uncorrected), but not the left hand (p = 0.74), indicating a DCZ-dependent behavioral deficit in the hand dominated by the DREADD-expressing hemisphere. Data are from monkey #2 examined over 5 independent experiments.

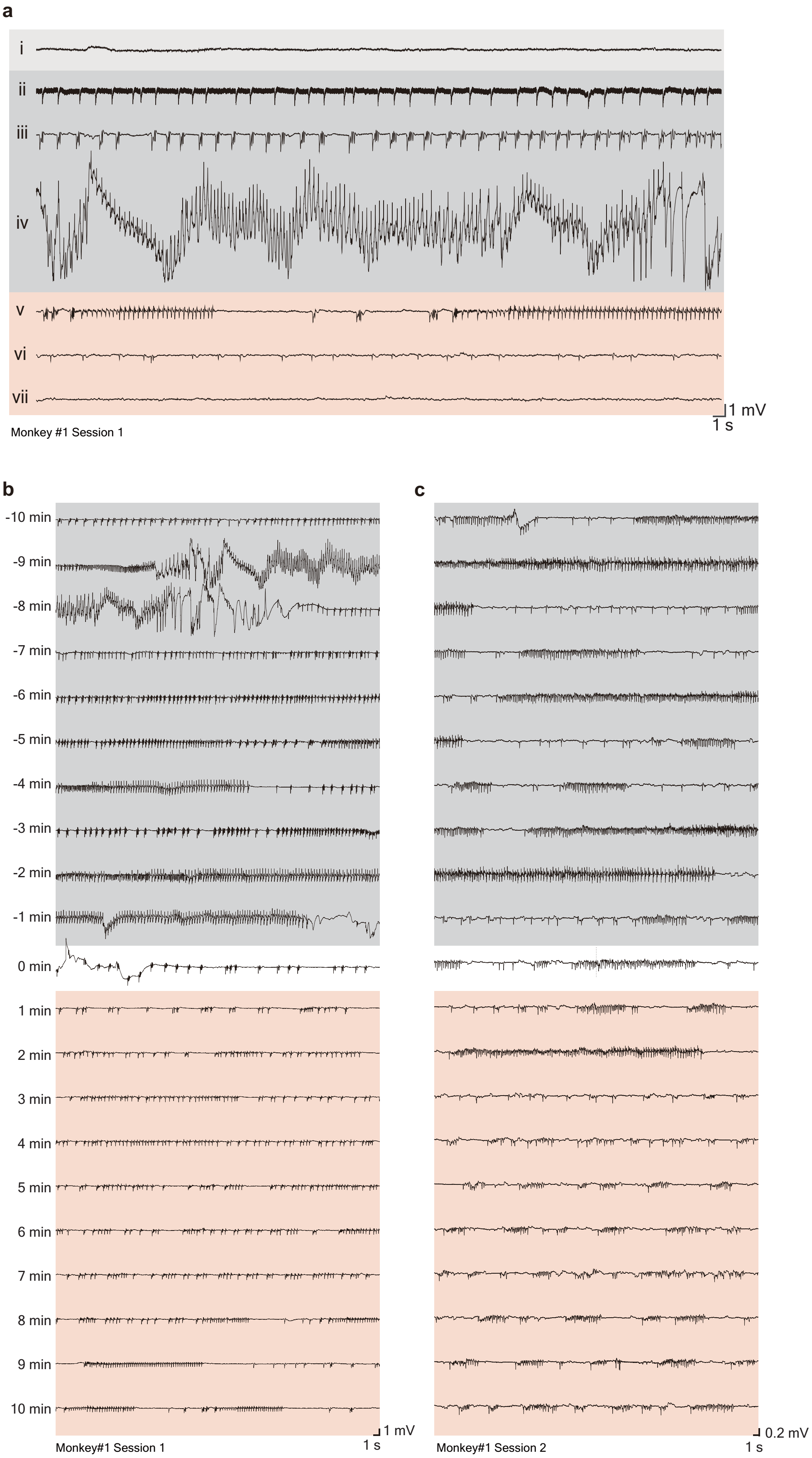
a
i
ii
iii
iv
v
vi
vii
1 mV
1 s
Monkey #1 Session 1
b
-10 min
-9 min
-8 min
-7 min
-6 min
-5 min
-4 min
-3 min
-2 min
-1 min
0 min
1 min
2 min
3 min
4 min
5 min
6 min
7 min
8 min
9 min
10 min
1 mV
1 s
Monkey#1 Session 1
c
0.2 mV
1 s
Monkey#1 Session 2

Supplementary Figure 3. Raw ECoG traces. a. Typical 60-s long ECoG traces, partially shown in Fig. 2a, depicting baseline (i), spikes (ii, vi), multi-spike-wave complexes (iii, v), a sustained multi-spike complex (status epilepticus, iv), and the return to baseline (vii). **b,c**. Continuous ECoG traces before (top panels) and after (lower panels) DCZ administration in sessions 1 (left) and 2 (right) in monkey #1.

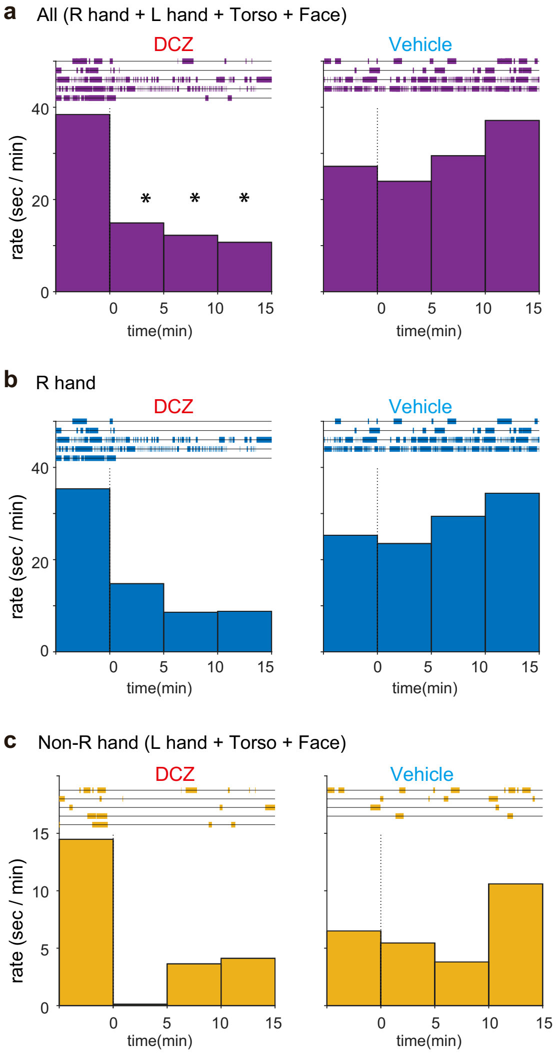

Supplementary Figure 4. **Effect of treatment on the frequency of distinct clonic seizures induced by bicuculline infusion in the left MI cortex.** Colored bars in the top panels of each plot indicate the time at which respective epileptic events were observed in each session. Histograms in the bottom panels depict the session-averaged rate of respective epileptic events within 5-min time windows. The rates of clonic seizures are shown for all observed body parts (**a**, All), the right hand (**b**, R hand), and body parts other than the right hand (**c**, Non-R hand). There was a significant effect of DCZ treatment (p = 0.0034, one-way ANOVA). Asterisks represent a significant difference (p = 0.030, 0.023, 0.038 respectively, post-hoc Dunnett's multiple comparison test) from pre-treatment baseline (−5 to 0 min time bin).

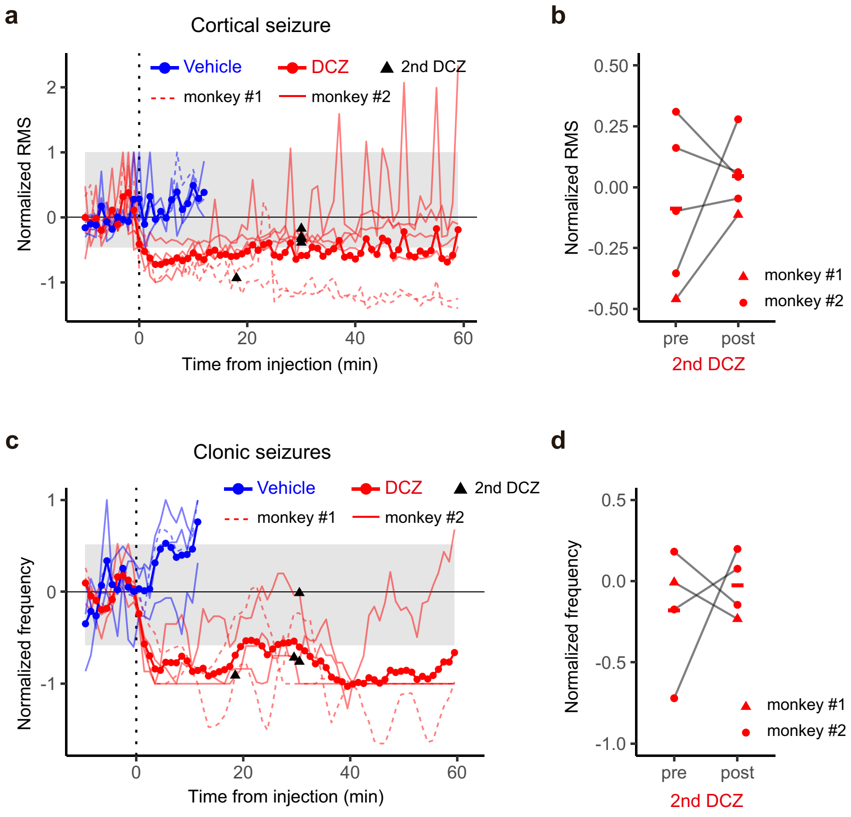

Supplementary Figure 5. Effect of DCZ administration on cortical and clonic seizures throughout a session. a. Changes in normalized seizure amplitude during a 1-h session (partially shown in Fig. 3a). Connected dots and lines represent mean and individual session amplitudes, respectively. Seizure amplitudes were normalized with respect to change from the average during the pre-treatment period, and scaled by the maximum values in the individual sessions. **b.** Impact of the second DCZ treatment on cortical seizure amplitude; no significant difference was observed between pre- (6 to 2 min before treatment) and post-treatment periods (2 to 6 min after treatment) (two-sided paired t-test, $p = 0.45$, $n = 2$ animals examined over 5 independent sessions). **c.** Changes in the normalized frequency of clonic seizures (mean ± standard error) in the hand/arm/torso/face over time after DCZ (red) and vehicle (blue) treatment during a 1-h session (partially shown in Fig. 3c). **d**. Impact of the second DCZ treatment on the frequency of clonic seizures; no significant difference was observed between pre- and post-treatment periods (two-sided paired t-test, $p = 0.62$, $n = 2$ animals examined over 4 independent sessions).

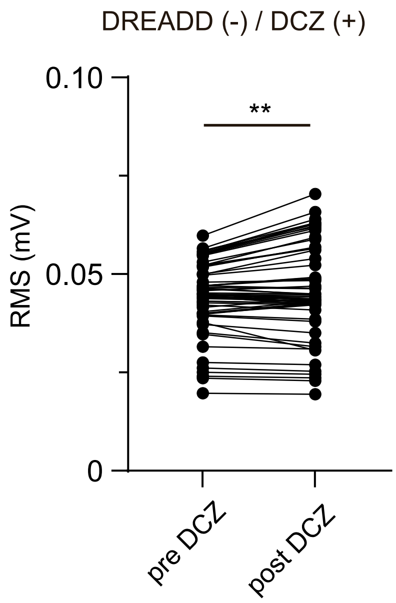

Supplementary Figure 6. DCZ treatment did not attenuate seizures in the contralateral (non-DREADD) hemisphere. Pre- and post-treatment seizure amplitudes at all ECoG channels were defined as the root-mean-square (RMS), averaged within 3-min time windows (pre-treatment: 4 to 1 minutes before; post-treatment: 3 to 6 minutes after). Data were recorded from the contralateral (non-DREADD) hemisphere of monkey #2 examined over 63 channels after bicuculline injection within the contralateral hemisphere. Seizure amplitude significantly increased after DCZ administration (two-sided paired t-test; $p = 0.0016$).

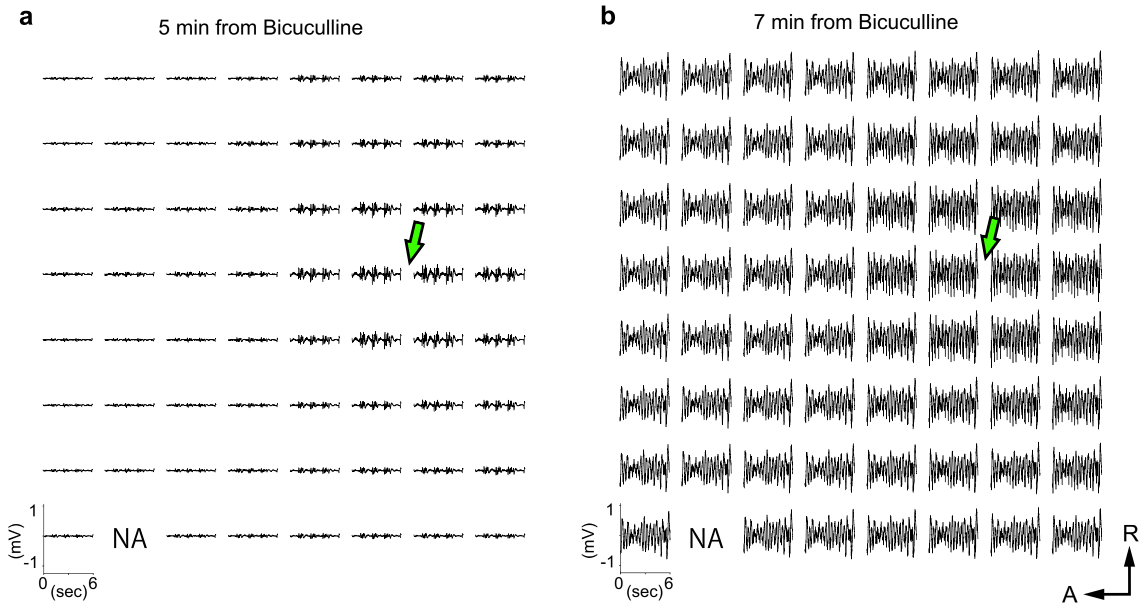

Supplementary Figure 7. Raw ECoG waveforms from monkey #1 after bicuculline infusion. a. A response pattern typical of focal seizures. **b.** A response pattern typical of wide-spread seizures. Green arrows depict the site of bicuculline local infusion. *NA*, Non-available recording channel.

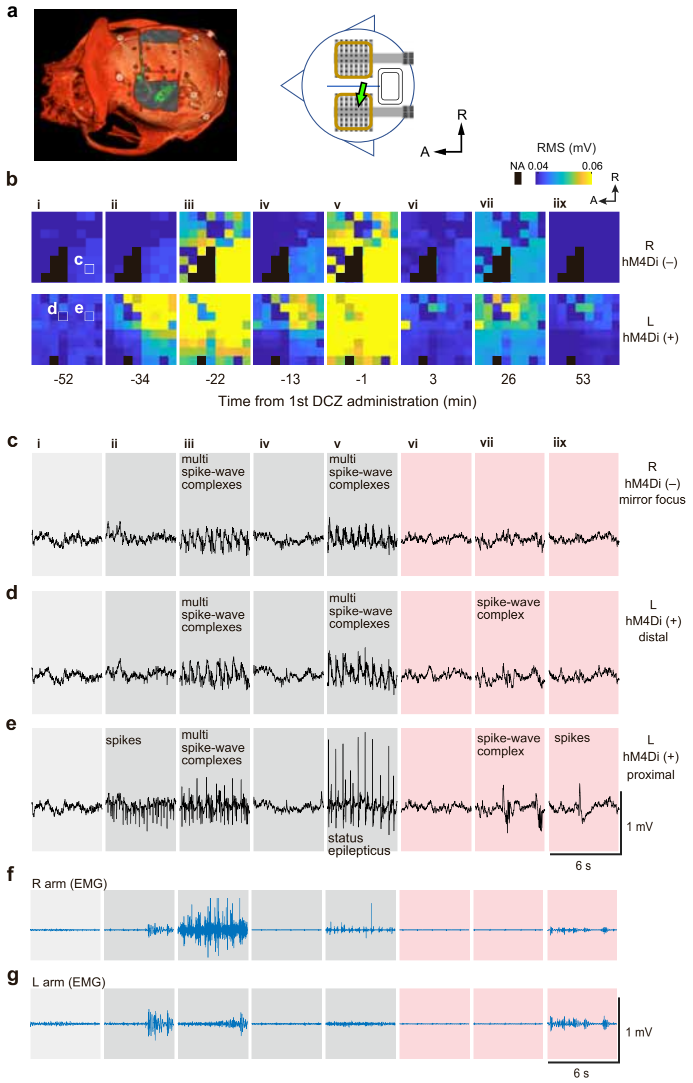

Supplementary Figure 8. Chemogenetic attenuation of focal-to-bilateral seizure. a. (Left) Location of the hM4Di expression (green) relative to the skull and sulci, visualized by superimposing CT, MR, and PET images. (Right) Schematic illustration of ECoG electrodes. Green arrows depict the site of bicuculline infusion. **b**. Example seizure-amplitude maps are shown as the root-mean-square (RMS) of the raw signals in a pseudo-color scale recorded from ECoGs in the non-DREADD (upper) and DREADD (lower) hemispheres. Black rectangles indicate non-available (NA) recording channels. **c-e**. Examples of raw ECoG signals showing typical waveforms before (**i**), after bicuculline infusion (**ii-v**), and after DCZ administration (**vi-iix**), recorded from the channels indicated in **b-i**. **f, g**. Examples of raw electromyography (EMG) signals recorded from right and left arms (brachioradialis muscles), respectively. Data are from session 4 in monkey #2.

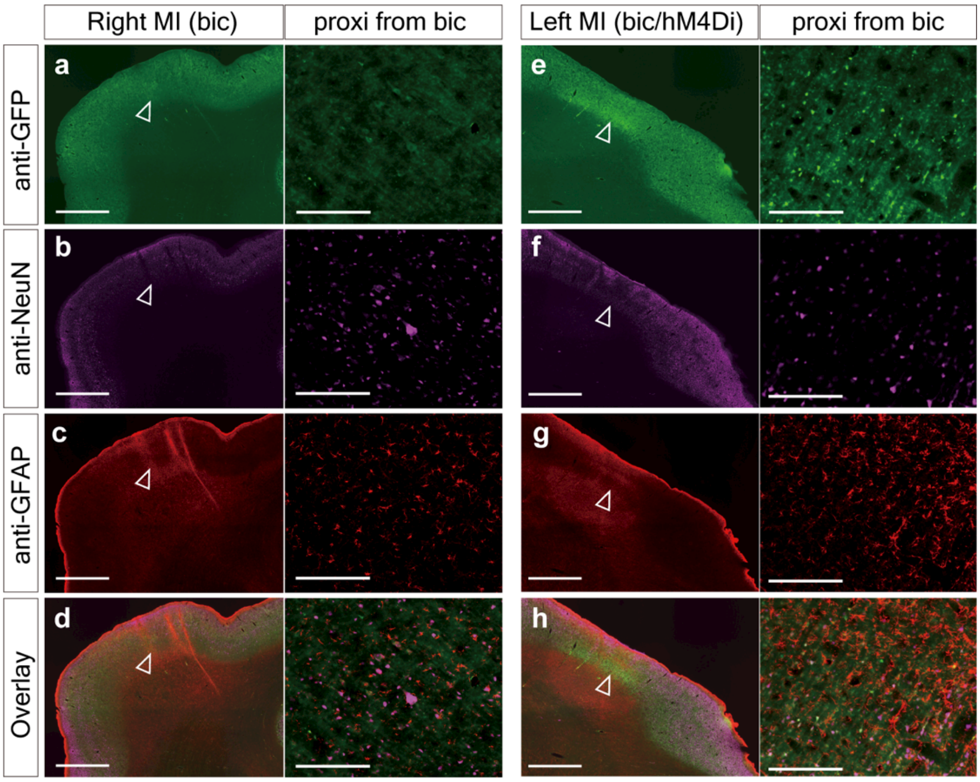

Supplementary Figure 9. Histological confirmation in monkey #2. **a-d.** Anti-GFP, Anti-NeuN, and Anti-GFAP immunofluorescent staining and overlay images in the right MI (non-DREADD hemisphere). Images were digitally enhanced by linearly redefining the tone curves. Open arrow heads in left panels point to the acquisition position of high-magnification images (right) proximal to the bicuculline infusion site. **e-h** same as **a-d** but for the left MI (DREADD hemisphere). Scale bars: left, 3 mm; right, 100 µm. Similar observations were made on monkey #1 (see Fig. 5)
