# Supplementary material for: Chemogenetic attenuation of cortical seizures in nonhuman primates
Source: Nat Commun. 2023 Feb 28;14:971. doi: 10.1038/s41467-023-36642-6 (PMC9975184; doi:10.1038/s41467-023-36642-6)
Supplement: Supplementary file 3 — Reporting Summary [file 41467_2023_36642_MOESM3_ESM.pdf]

nature portfolio

Corresponding author(s): Naohisa Miyakawa
Takafumi Minamimoto

Last updated by author(s): Jan 20, 2023

Reporting Summary

Nature Portfolio wishes to improve the reproducibility of the work that we publish. This form provides structure for consistency and transparency in reporting. For further information on Nature Portfolio policies, see our Editorial Policies and the Editorial Policy Checklist.

Statistics

For all statistical analyses, confirm that the following items are present in the figure legend, table legend, main text, or Methods section.

n/a	Confirmed	
☐	☒	The exact sample size (*n*) for each experimental group/condition, given as a discrete number and unit of measurement
☐	☒	A statement on whether measurements were taken from distinct samples or whether the same sample was measured repeatedly
☐	☒	The statistical test(s) used AND whether they are one- or two-sided<br>*Only common tests should be described solely by name; describe more complex techniques in the Methods section.*
☐	☒	A description of all covariates tested
☐	☒	A description of any assumptions or corrections, such as tests of normality and adjustment for multiple comparisons
☐	☒	A full description of the statistical parameters including central tendency (e.g. means) or other basic estimates (e.g. regression coefficient) AND variation (e.g. standard deviation) or associated estimates of uncertainty (e.g. confidence intervals)
☐	☒	For null hypothesis testing, the test statistic (e.g. *F*, *t*, *r*) with confidence intervals, effect sizes, degrees of freedom and *P* value noted<br>*Give P values as exact values whenever suitable.*
☐	☒	For Bayesian analysis, information on the choice of priors and Markov chain Monte Carlo settings
☒	☐	For hierarchical and complex designs, identification of the appropriate level for tests and full reporting of outcomes
☒	☐	Estimates of effect sizes (e.g. Cohen's *d*, Pearson's *r*), indicating how they were calculated

Our web collection on statistics for biologists contains articles on many of the points above.

Software and code

Policy information about availability of computer code

Data collection	Data for each experiment was collected using<br>PET: microPET Manager 2.8 (Siemens)<br>Electrophysiology: System3 (TOT)<br>Immunohistochemistry: BZ-X Viewer v.1.03 (Keyence) and NanoZoomer S60 (Hamamatsu Photonics K.K)
Data analysis	Data were analyzed using the follwing softwares:<br>PET: PMOD 3.7 (PMOD Technologies) for [C-II]DCZ, SPM12 (Wellcome Centre for Human Neuroimaging, UCL) and MATLAB R2016a (Math Works)<br>Electrophysiology: OpenDeveloper(TDT) and MATLAB R2018b (MathWorks)<br>Other analyses were performed with Prism 9 (GraphPad), MATLAB R2018b, or R statistical computing environment (version 4.0.3) with R packages (CmdStan ver. 2.30.1 and cmdstanr ver. 0.5.2).

For manuscripts utilizing custom algorithms or software that are central to the research but not yet described in published literature, software must be made available to editors and reviewers. We strongly encourage code deposition in a community repository (e.g. GitHub). See the Nature Portfolio guidelines for submitting code & software for further information.

Data

Policy information about availability of data

All manuscripts must include a data availability statement. This statement should provide the following information, where applicable:

- Accession codes, unique identifiers, or web links for publicly available datasets
- A description of any restrictions on data availability
- For clinical datasets or third party data, please ensure that the statement adheres to our policy

The source data generated in this study have been deposited in the Open Science Framework database (http:/ /github.com/minamimoto-lab/2022-Miyakawa-epilepsy).

Human research participants

Policy information about studies involving human research participants and Sex and Gender in Research.

Reporting on sex and gender	NA
Population characteristics	NA
Recruitment	NA
Ethics oversight	NA

Note that full information on the approval of the study protocol must also be provided in the manuscript.

Field-specific reporting

Please select the one below that is the best fit for your research. If you are not sure, read the appropriate sections before making your selection.

[x] Life sciences [ ] Behavioural & social sciences [ ] Ecological, evolutionary & environmental sciences

For a reference copy of the document with all sections, see nature.com/documents/nr-reporting-summary-flat.pdf

Life sciences study design

All studies must disclose on these points even when the disclosure is negative.

Sample size	The number of animal used was two, which is a standard of typical neurophysiological study using nonhuman primates. No statistical methods were used to pre-determine sample sizes. We determined the sample size based on those reported in typical monkey neurophysiology experiments, which provides enough power to validate claims.
Data exclusions	No samples were excluded from the analysis.
Replication	In all experiments, at least 2 subjects were used except for control behavioral experiments (Fig. 52). Results were consistent across subjects, suggesting reproducibility of the results.
Randomization	In behavioral experiments, the order of tested hands (ipsilateral VS contralateral) was randomized.
Blinding	Blinding was not used for data collection in the animal experiments as they were automated. Scientific rigor is provided by replication across multiple animals and paradigms (electrophysiology, PET, and behavior). Regarding data analysis, the same analysis were performed on control data. No further blinding during the analysis was implemented.

Reporting for specific materials, systems and methods

We require information from authors about some types of materials, experimental systems and methods used in many studies. Here, indicate whether each material, system or method listed is relevant to your study. If you are not sure if a list item applies to your research, read the appropriate section before selecting a response.

Materials & experimental systems

n/a	Involved in the study	
	x	Antibodies
x		Eukaryotic cell lines
x		Palaeontology and archaeology
	x	Animals and other organisms
x		Clinical data
x		Dual use research of concern

Methods

n/a	Involved in the study	
x		ChIP-seq
x		Flow cytometry
x		MRI-based neuroimaging

Antibodies

Antibodies used	GFP Recombinant Rabbit Monoclonal Antibody, invitrogen (Thermo Fisher Scientific), Cat: G10362, Lot.2180285<br>Biotin SP Donkey Anti Rabbit IgG, Jackson, Cat. 711-065-152, Lot.140405<br>GFP: Origin R1091P, Anti-GFP Goat Polyclonal Antibody, lot 35578<br>lbal: Wako 019-19741, Anti-lbal Rabbit Polyclonal Antibody, lot CAE1308 or SKQ2227<br>CD8: Bio-Rad MCA4609T, Anti-human CD8 Mouse Monoclonal Antibodty, lot 070714<br>GFAP: SIGMA-ALDRICH, G3893, Anti-GFAP mouse Monoclonal Antibody, lot 107M4792V<br>NeuN: MILLIPORE, ABN90, Anti-NeuN gunea-pig polyclonal Antibody), lot 3660324<br>Alexa 488-conjugated donkey anti-goat IgG antibody, Invitrogen<br>Alexa 555-conjugated donkey anti-rabbit IgG antibody, Invitrogen<br>Alexa 647-conjugated donkey anti-mouse IgG antibody, Invitrogen
Validation	All commercial antibodies were validated by manufacturer, as follows:<br>rabbit anti-G FP monoclonal antibody: https:/ /www.thermofisher.com/anti body /product/G FP-Antibody-Recombinant -Monoclonal/G10362<br>Biotin SP Donkey Anti-Rabbit IgG: https://www.jacksonimmuno.com/catalog/products/711-065-152 https://labchem-wako.fujifilm.com/us/product/detail/WOI WOIOI-197 4. html<br>https://bio-rad-anti body.jp/antibody /detail/gid :bio-rad/cnu m :M CA4609T /<br>https://www.sigmaaldrich.com/」 P/ja/product/sigma/g3893<br>https://www.merckmillipore.com/」 P/ja/product/Anti-NeuN-Antibody,MM_NF-ABN90

Animals and other research organisms

Policy information about studies involving animals; ARRIVE guidelines recommended for reporting animal research, and Sex and Gender in Research

Laboratory animals	Two male cynomolgus macaque monkeys (Macaca fascicularis; 5 yrs old 4.8 kg, and 6 yrs old 5.4 kg) were used for the experiments. The animals were provided by HAMRI Co., Ltd., Japan.
Wild animals	No wild animals were used.
Reporting on sex	Two male macaque monkeys were used but no females were involved.
Field-collected samples	No field-collected samples were used.
Ethics oversight	All experimental procedures involving animals were carried out in accordance with the Guide for the Care and Use of Nonhuman primates in Neuroscience Research (The Japan Neuroscience Society; https://www.jnss.org/en/animal_primates) and were approved by the Animal Ethics Committee of the National Institute of Quantum Sciences and Technology.

Note that full information on the approval of the study protocol must also be provided in the manuscript.
